# Predictors of length of hospital stay after pediatric Ebstein anomaly corrective surgery: a retrospective cohort study

**DOI:** 10.1186/s12887-024-04936-3

**Published:** 2024-08-10

**Authors:** Qiao Liu, Xie Wu, Yinan Li, Jie Ding, Hongbai Wang, Dou Dou, Ran An, Dongyun Bie, Yuan Jia, Su Yuan, Fuxia Yan

**Affiliations:** 1Department of Anesthesiology, Fuwai Hospital, National Center of Cardiovascular Diseases, Chinese Academy of Medical Sciences and Peking Union Medical College, 167 Beilishi Road, Xicheng District, Beijing, 100037 China

**Keywords:** Ebstein anomaly (EA), Time to hospital discharge (THD), Pediatric cardiac surgery, Predictor

## Abstract

**Background:**

The remarkable advancements in surgical techniques over recent years have shifted the clinical focus from merely reducing mortality to enhancing the quality of postoperative recovery. The duration of a patient’s hospital stay serves as a crucial indicator in evaluating postoperative recovery and surgical outcomes. This study aims to identify predictors of the length of hospital stay for children who have undergone corrective surgery for Ebstein Anomaly (EA).

**Methods:**

We conducted a retrospective cohort study on children (under 18 years of age) diagnosed with EA who were admitted for corrective surgery between January 2009 and November 2021 at Fuwai Hospital. The primary outcome was the Time to Hospital Discharge (THD). Cox proportional hazard models were utilized to identify predictors of THD. In the context of time-to-event analysis, discharge was considered an event. In cases where death occurred before discharge, it was defined as an extended THD, input as 100 days (exceeding the longest observed THD), and considered as a non-event.

**Results:**

A total of 270 children were included in this study, out of which three died in the hospital. Following the Cox proportional hazard analysis, six predictors of THD were identified. The hazard ratios and corresponding 95% confidence intervals were as follows: age, 1.030(1.005,1.055); C/*R* > 0.65, 0.507(0.364,0.707); Carpentier type C or D, 0.578(0.429,0.779); CPB time, 0.995(0.991,0.998); dexamethasone, 1.373(1.051,1.795); and transfusion, 0.680(0.529,0.875). The children were categorized into three groups based on the quartile of THD. Compared to children in the ≤ 6 days group, those in the ≥ 11 days group were associated with a higher incidence of adverse outcomes. Additionally, the duration of mechanical ventilation and ICU stay, as well as hospital costs, were significantly higher in this group.

**Conclusion:**

We identified six predictors of THD for children undergoing corrective surgery for EA. Clinicians can utilize these variables to optimize perioperative management strategies, reduce adverse complications, improve postoperative recovery, and reduce unnecessary medical expenses.

**Supplementary Information:**

The online version contains supplementary material available at 10.1186/s12887-024-04936-3.

## Background

Accurate prediction of hospitalization duration is crucial for hospital management and rational allocation of medical resources, which helps to improve the quality and efficiency of medical care services and avoid unnecessary long-term care. Postoperative hospital stay duration is one of the significant indicators in pediatric cardiac surgery and is associated with long-term neurological development after surgery [1, 2]. Even a single additional day in the hospital can significantly impact morbidity and hospital costs following cardiac surgery. In clinical practice, the preoperative status and perioperative information of patients are important sources for predicting the length of hospital stay. Previous studies [3, 4] have analyzed the impact of relevant clinical variables on the length of hospital stay through scientific statistical models and large data sets, helping hospital administrative departments plan personnel allocation reasonably. Ebstein Anomaly (EA), also known as tricuspid valve displacement malformation, is a rare congenital heart disease characterized by the displacement of the posterior and septal leaflets of the tricuspid valve to the right ventricle, resulting in an atrialized right ventricle (aRV) [5]. Patients with EA usually combined with patent foramen ovale or atrial septal defects and exhibit varying degrees of tricuspid valve insufficiency and right heart dysfunction [6]. The primary goal of EA corrective surgery is to restore the physiological function of the tricuspid valve and the right ventricle. Although EA is a rare and complex congenital heart disease with an incidence as low as 1 per 200,000 live births [6, 7], focusing on patients with rare diseases can still yield valuable insights. Therefore, this study sought to identify predictors of the length of hospital stay in children who underwent EA corrective surgery by retrospectively collecting perioperative clinical information.

## Methods

This study was designed as a retrospective cohort study and adheres to the Strengthening the Reporting of Observational Studies in Epidemiology (**STROBE**) statement (Additional File 1). All methods were compliant with our hospital regulations and the 1964 Helsinki Declaration. The research was approved by the Ethics Committee of the Chinese Academy of Medical Sciences Fuwai Hospital (No.2022 − 1647), and informed consent was waived due to the nature of the retrospective study. We included children (age < 18 years) who underwent EA corrective surgery in Fuwai Hospital from January 2009 to November 2021. Children combined with confounding factors that may affect the hospital stay time were excluded: previous history of bidirectional glenn surgery, complex cardiac malformations (corrected transposition of a great artery, pulmonary atresia, single ventricle, endocardial cushion defect, coronary artery fistula). The surgical indications for EA patients are as follows: severe TR, cyanosis, dyspnea, right heart failure, cardiac hypertrophy, C/*R* > 0.65, tachyarrhythmias, and associated other cardiac defects [8]. Surgeons performed cardiac surgery for EA children based on the cardiac anatomical structure and clinical experience. The surgical technique included modified Carpentier’s method, modified Danielson method, tricuspid valve replacement, surgical ablation or division, and radiofrequency ablation.

Children’s data was collected from admission to discharge via the institution’s electronic medical record system and checked by two independent researchers. The preoperative information was obtained: age, gestational age, sex, weight, height, body mass index (BMI), blood oxygen saturation (SpO2), New York Heart Association Classification, symptoms, medication, cardiothoracic ratio (C/R), arrhythmia, cardiac defects, preoperative laboratory tests, and echocardiography data. The SpO2 was obtained before surgery when the children inhaled the air. A bluish or purplish discoloration of the skin, lips, or nail beds and a SpO_2_ less than 90% after inhaling air was considered cyanosis. The intraoperative data included surgical technique, cardiopulmonary bypass (CPB) time, aortic cross-clamp (ACC) time, the minimum temperature during CPB, drugs, the intraoperative maximum vasoactive-inotropic score (VIS_m_), infusion volume, transfusion, and blood loss. Clinicians used the Philips iE33 ultrasound system (Philips Medical Systems, Andover, MA) with S8 or S12 phased array probe to evaluate the preoperative cardiac structure of EA patients in our hospital. The acquisition of echocardiography images through multiple standard ultrasound windows, including the parasternal long axis, parasternal short axis, apical four chambers, and subcostal view. We collected the following parameters in particular: left ventricular ejection fraction, tricuspid regurgitation (TR), right ventricular anteroposterior diameter, septal leaflets displacement, posterior leaflets displacement, aortic valve annulus diameter, pulmonary valve annulus diameter, left pulmonary artery diameter, and right pulmonary artery diameter. TR was characterized as none, mild, moderate, and severe based on color flow maps. The Carpentier’s classification method was used to classify the anatomy of EA. In addition, the left ventricular end-diastolic diameter z-score (LVEDDz) was calculated to reflect the left ventricular end-diastolic diameter.

The primary outcome was the Time to Hospital Discharge (THD), defined as the number of days from surgery to discharge. Secondary outcomes included adverse events, mechanical ventilation time, acute kidney injury (AKI), acute hepatic injury (AHI), ICU stay time, hospital stay time, and hospitalization costs. Adverse events were a composite outcome referring to in-hospital death, re-exploratory thoracotomy, extracorporeal membrane oxygenation placement, reintubation, respiratory failure, low cardiac output syndrome (LCOS), malignant ventricular arrhythmia, and continuous renal replacement therapy. LCOS was defined as a maximum vasoactive inotropic score greater than 20 within 24 h after surgery [9]. Respiratory failure was defined as a postoperative oxygenation index of less than 100 [10]. AKI was defined as a postoperative creatinine level more than 1.5 times the baseline level [11]. AHI was defined as postoperative aspartate aminotransferase (AST) or alanine aminotransferase more than 2 times the upper limit, excluding increased AST within 24 h [12].

### Statistical analysis

To compare perioperative information of children with different hospital stay times, we divided them into three groups based on quartiles of THD. Descriptive statistics were used to summarize the children’s characteristics. Continuous data were presented as median (25th, 75th percentile) and compared using the Mann-Whitney U test (for two groups) or the Kruskal-Wallis H test (for more than two groups). Categorical variables were described as absolute counts with percentages and compared using the Chi-squared test. For the time-to-event analysis, hospital discharge was considered an event. In contrast, death before discharge was defined as a prolonged THD, input as 100 days (longer than the longest THD), and considered as no event. Univariable and multivariable COX proportional hazard models were used to assess predictors for THD. Variables with *P* < 0.1 in the univariable model and clinically relevant variables were incorporated into a multivariable model. Collinearity relationships between predictors were evaluated by tolerance and Variance Inflation Factor (VIF). Tolerance < 0.1 and/or VIF > 10 indicated a collinear relationship between variables. Results from the Cox proportional hazards models are presented as hazard ratios (HRs) with 95% confidence intervals (95% CIs). HR < 1 indicated a lower discharge rate (longer THD), and HR > 1 indicated a higher discharge rate (shorter THD). The Kaplan‑Meier survival curves were used to present the relationships between predictors and postoperative hospital stay time. *P* < 0.05 was considered statistically significant. All statistical analysis was conducted in SPSS software version 25 (IBM, Armonk, NY, USA) and GraphPad Prism 7.0 (GraphPad Software, Inc.).

## Results

### Basic characteristics

Over the past 13 years, 278 children underwent EA corrective surgeries at Fuwai Hospital. Eight children were excluded due to previous history of bidirectional glenn surgery(*n* = 4), and complex cardiac malformations (*n* = 6). Consequently, 270 children were included in this study. Children were classified into three groups according to the THD quartile and the perioperative information was displayed in Tables 1 and 2. The median age of all children was 5.2(2.5,10.7) years, and 146(54.1%) were male. The median SpO2 was 98% (95%,99%), and most children combined with a heart murmur (72.6%). 19.6% (*n* = 53) of children had a C/*R* > 0.65 and 36 (13.3%) children with preoperative WPW syndrome. Most children had an atrial septal defect or patent foramen ovale. Regarding echocardiography results, the median ejection fraction was 65% (62%,70%), the median LVEDDz was − 1.9(-2.8, -1), the median displacement of septal leaflet and posterior leaflet were 20(14,27) and 32(12,42), respectively. 180(66.7%) children presented with severe TR. Intraoperative data showed that 205(75.9%) surgeries performed modified Carpentier’s technique, the median CPB and aortic cross-clamp times were 110 (90,139) and 76 (61,96) minutes, respectively. The median VIS_m_ was 9.5(5,13), and 148(54.8%) children received transfusions. Children in the ≥ 11 days group were younger, and had a lower value of BMI, hemoglobin, hematocrit, and aortic annular diameter, compared to those in the ≤ 6 days group. They also had higher VISm, longer CPB time, and a higher rate of C/*R* > 0.65, Carpentier C or D, glenn, and transfusion.

**Table 1:** The demographic and preoperative information of the study children

Variable	Total	≤ 6 days	7 to 10 days	≥ 11 days	*P* value
Age (years)	5.2(2.5,10.7)	6.2(3,13.8)	6(2.9,10.8)	4(1.9,6.7)	0.022^b, c^
Gestational age (weeks)	40(39,40)	40(40,40)	40(39,40)	40(39,40)	0.189
male	146(54.1%)	33(63.5%)	80(55.2%)	33(45.2%)	0.041^c^
Weight (kg)	18.8(13,33)	22.3(14.4,53.3)	19(13.8,35)	16(10.5,24.5)	0.005^b, c^
Height (cm)	110(89,141)	117(92,161)	112(92,142)	104(83,122)	0.010 ^b, c^
BMI (kg/m^2^)	16.5(15.1,18.4)	17.6(16,21.4)	16.4(14.9,18.1)	16(14.6,17.8)	< 0.001^a, b^
SpO2(%)	98(95,99)	98(96,99)	98(95,99)	96(91,99)	0.138
NYHA class (III/IV)	32(11.9%)	4(7.7%)	13(9%)	15(20.5%)	0.018^b, c^
**Symptoms**					
Cyanosis	46(17%)	5(9.6%)	28(19.3%)	13(17.8%)	0.290^b^
Tachypnoea	20(7.4%)	4(7.7%)	7(4.8%)	9(12.3%)	0.237^c^
Heart murmur	196(72.6%)	36(69.2%)	106(73.1%)	54(74%)	0.579
Palpitation	21(7.8%)	8(15.4%)	11(7.6%)	2(2.7%)	0.010^a, b,c^
Chest tightness	9(3.3%)	2(3.8%)	5(3.4%)	2(2.7%)	0.726
**Medication**					
Diuretics	105(38.9%)	22(42.3%)	55(37.9%)	28(38.4%)	0.352
Beta-blocker	8(3%)	1(1.9%)	4(2.8%)	3(4.1%)	0.465
ACEI	18(6.7%)	1(1.9%)	11(7.6%)	6(8.2%)	0.195^b^
Inotropes	19(7%)	4(7.6%)	12(8.2%)	3(4.2%)	0.574
C/*R* > 0.65	53(19.6%)	8(15.4%)	21(14.5%)	24(32.9%)	0.007^b, c^
WPW syndrome	36(13.3%)	8(15.4%)	20(13.8%)	8(11%)	0.459
ASD	83(37.7%)	19(38.8%)	41(35%)	23(42.6%)	0.668
PDA	4(1.8%)	0(0%)	1(0.95)	3(5.6%)	0.032^b, c^
PFO	11(22.4%)	40(34.2%)	17(31.5%)	68(30.9%)	0.343
WCC (10^9^/L)	8.3(6.5,9.5)	8(6.8,9.5)	8.3(6.3,9.4)	8.3(6.7,10.1)	0.766
RBC (10^12^/L)	4.8(4.6,5.2)	4.8(4.6,5.3)	4.8(4.6,5.1)	4.8(4.5,5)	0.153
PLT (10^9^/L)	279(239,333)	305(259,345)	279(242,329)	279(228,319)	0.172
Neutrophil	3.1(2.5,4.2)	3.6(2.4,4.6)	3.1(2.7,4.2)	2.7(2.4,4.2)	0.184
Lymphocyte	3.9(2.9,4.7)	3.6(2.8,5)	3.7(2.8,4.6)	4.5(3.4,4.6)	0.107
Monocyte	0.48(0.37,0.58)	0.47(0.35,0.58)	0.47(0.36,0.58)	0.51(0.38,0.58)	0.474
Hb(g/L)	135(128,144)	140(129,150)	136(128,145)	134(126,139)	0.003^b, c^
Hct (%)	39.4(36.8,42.4)	41.2(38.2,44.1)	39.4(36.8,42.5)	37.6(36.8,40.5)	< 0.001^b, c^
CK-MB (IU/L)	16(9,27)	14(5.8,22.8)	19(12,28)	13(3.3,25.5)	0.016^a, c^
hs-CRP(mg/L)	0.41(0.18,0.95)	0.41(0.14,1.08)	0.41(0.17,0.94)	0.41(0.28,0.95)	0.902

Quantitative variables are expressed as median with interquartile range; Categorical variables are expressed as frequency (percentage). BMI, body mass index; SpO2, peripheral oxygen saturation; NYHA class, New York Heart Association Classification; ACEI, angiotensin-converting enzyme inhibitor; C/R, cardiothoracic ratio; WPW, Wolff-Parkinson-White; ASD, atrial septal defect; PDA, patent ductus arteriosus; PFO, patent foramen ovale; WCC, white blood cell; RBC, red blood cell; PLT, platelet; Hb, Hemoglobin; Hct, hematocrit; CK-MB, isoenzyme of creatine kinase-MB; hs-CRP, C-reactive protein; a: the significant difference of results between the ≤ 6 days group and the 7 to 10 days group; b: the significant difference of results between the ≤ 6 days group and the ≥ 11 days group; c: the significant difference of results between 7 to 10 days group and ≥ 11 days group

**Table 2:** The cardiac diagnosis and intraoperative information of the study children

Variable	Total	≤ 6 days	7 to 10 days	≥ 11 days	*P* value
**Echocardiography data**					
LVEF (%)	65(62,70)	65(61,70)	65(62,70)	66(62,71)	0.680
TR					0.270
Mild	20(7.4%)	1(1.9%)	13(9%)	6(8.2%)	
Moderate	53(19.6%)	10(19.2%)	30(20.7%)	13(17.8%)	
Severe	180(66.7%)	39(75%)	92(63.4%)	49(67.1%)	
Carpentier type					< 0.001^b, c^
Type A	38(14.1%)	12(23.1%)	20(13.8%)	6(8.2%)	
Type B	165(61.1%)	34(65.4%)	97(66.9%)	34(46.6%)	
Type C	62(23%)	6(11.5%)	28(19.3%)	28(38.4%)	
Type D	5(1.9%)	0(0%)	0(0%)	5(1.9%)	
LVEDDz	-1.9(-2.8,-1)	-1.7(-3.1,-0.9)	-2(-2.6,-1.2)	-1.9(-2.7,-1)	0.909
RVAD (mm)	25(20,30)	25(19,33)	25(19,30)	25(21,30)	0.768
SLD (mm)	20(14,27)	20(14,28)	20(14,26)	22(14,29)	0.973
PLD (mm)	32(12,42)	32(19,45)	32(22,43)	32(20,40)	0.781
ANND (mm)	15(13,18)	16(13,19)	15(13,18)	13(12,16)	0.001^b, c^
PVA (mm)	15(12,17.3)	16(12,18.8)	15(12,17)	14(11,17)	0.331
LPA (mm)	10(8,10)	10(8,11)	10(8,11)	9(7.5,10)	0.081
RPA (mm)	10(8,11)	10(8,11.8)	10(8,11)	9(7,10)	0.049^b^
**Surgical information**					
Surgical technique					
Modified Carpentier method	205(75.9%)	39(75%)	106(73.1%)	60(82.2%)	0.288
Modified Danielson method	63(23.3%)	13(25%)	38(26.2%)	12(16.4%)	0.210
TVR	9(3.3%)	4(7.7%)	1(0.7%)	4(5.5%)	0.726^a^
Glenn	50(18.5%)	3(5.8%)	28(19.3%)	19(26%)	0.005^ab^
Surgical ablation or division	5(1.9%)	1(1.9%)	2(1.4%)	2(2.7%)	0.684
Radiofrequency ablation	6(2.2%)	1(1.9%)	4(2.8%)	1(1.4%)	0.776
CPB time (min)	110(90,139)	101(89,134)	104(87,135)	129(105,147)	0.001^b, c^
ACC time (min)	76(61,96)	74(63,97)	71(60,92)	85(69,105)	0.034^b, c^
T_min_ (℃)	30(29.6,31)	30.5(29.6,31)	30(29.7,31)	30(29.3,31)	0.378
Sufentanil (ug/kg)	2.6(1.9,4.4)	2.6(1.7,4.9)	2.6(1.9,4.4)	2.5(1.9,4)	0.954
Dexamethasone	177(65.6%)	34(65.4%)	106(73.1%)	37(50.7%)	0.042^b, c^
Ulinastatin	92(34.1%)	19(36.5%)	49(33.8%)	24(32.9%)	0.683
VIS_m_	9.5(5,13)	6(5,10)	9(5,13)	10(6,16)	0.006^b, c^
Infusion volume (ml)	90(55,300)	100(56,300)	90(55,300)	80(58,200)	0.428
Transfusion	148(54.8%)	20(38.5%)	81(55.9%)	47(64.4%)	0.005^b^
Blood loss (ml)	60(30,200)	70(30,360)	70(30,200)	45(23,80)	0.020^c^

Quantitative variables are expressed as median with interquartile range; Categorical variables are expressed as frequency (percentage). LVEF, left ventricular ejection fraction; TR, tricuspid regurgitation; LVEDDz, left ventricular end-diastolic diameter z-score; RVAD, right ventricular anteroposterior diameter; SLD, Septal leaflets displacement; PLD, Posterior leaflets displacement; ANND, aortic valve annulus; PVAD, pulmonary valve annulus diameter; LPA, left pulmonary artery diameter; RPA, right pulmonary artery diameter; TVR, tricuspid valve replacement; CPB, cardiopulmonary bypass; ACC, aortic cross-clamp; T_min_, the minimum temperature; VIS_m_, the intraoperative maximum vasoactive inotropic score; a: the significant difference of results between the ≤ 6 days group and the 7 to 10 days group; b: the significant difference of results between the ≤ 6 days group and the ≥ 11 days group; c: the significant difference of results between 7 to 10 days group and ≥ 11 days group

### Predictors of THD

**Fig. 1:**
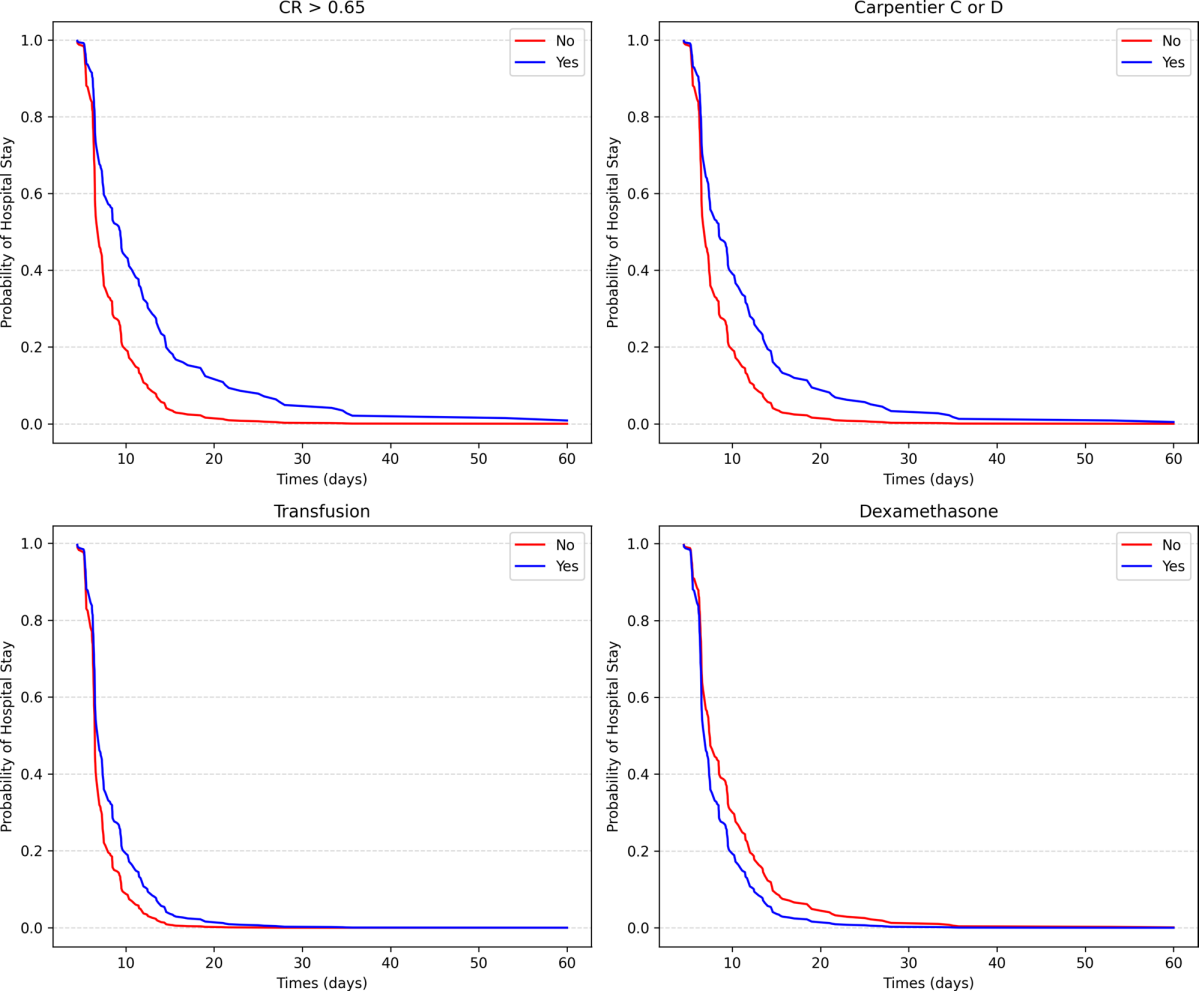
The Kaplan‑Meier survival curves for postoperative hospitalization. Times refers to the length of hospital stay after surgery. C/R, cardiothoracic ratio

The univariable analysis demonstrated that NYHA class (III/IV), C/*R* > 0.65, Carpentier type C or D, glenn, transfusion, higher CPB time, and VIS_m_ were associated with a lower rate of hospital discharge (i.e., longer THD). In contrast, older age, longer height, palpitation, and dexamethasone were associated with a higher rate of hospital discharge (i.e., shorter THD). The HRs and 95% CIs of univariable analysis were shown in Additional File 2. A multicollinearity test was conducted and found a highly collinear relationship between age and height. Given that height was correlated with age, and then we excluded height from the multivariable analysis. Upon performing a multivariable Cox analysis (Table 3), C/*R* > 0.65, Carpentier type C or D, longer CPB time, and transfusion were still significantly associated with a lower rate of hospital discharge (i.e., longer THD). In contrast, older age and the use of dexamethasone were associated with a higher rate of hospital discharge (i.e., shorter THD). The Kaplan‑Meier survival curves from the multivariate Cox analysis also displayed the relationship of categorical variables (C/*R* > 0.65, Carpentier type C or D, transfusion, and dexamethasone) with postoperative hospitalization (Fig. 1).

**Table 3:** Multivariable cox proportional hazards model

Predictor	Univariable model		Multivariable model	
	HR(95%CI)	*P* value	HR(95%CI)	*P* value
Age (years)	1.032(1.008,1.057)	0.009	1.030(1.005,1.055)	0.019
Height (cm)	1.005(1.002,1.009)	0.003		
NYHA class (III/IV)	0.651(0.448,0.945)	0.024		
Palpitation	1.984(1.264,3.112)	0.003		
C/*R* > 0.65	0.494(0.357,0.682)	< 0.001	0.507(0.364,0.707)	< 0.001
Carpentier type C or D	0.477(0.356,0.638)	< 0.001	0.578(0.429,0.779)	< 0.001
Glenn	0.638(0.467,0.871)	0.005		
CPB time (min)	0.995(0.992,0.998)	0.001	0.995(0.991,0.998)	0.001
Dexamethasone	1.494(1.156,1.930)	0.002	1.373(1.051,1.795)	0.020
VIS_m_	0.983(0.966,1.000)	0.046		
Transfusion	0.698(0.546,0.892)	0.004	0.680(0.529,0.875)	0.003

HR, hazard ratio; CI, confidence interval; NYHA class, New York Heart Association Classification; C/R, cardiothoracic ratio; CPB, cardiopulmonary bypass; VIS_m_, the intraoperative maximum of vasoactive inotropic score

### In-hospital outcomes

**Fig. 2:**
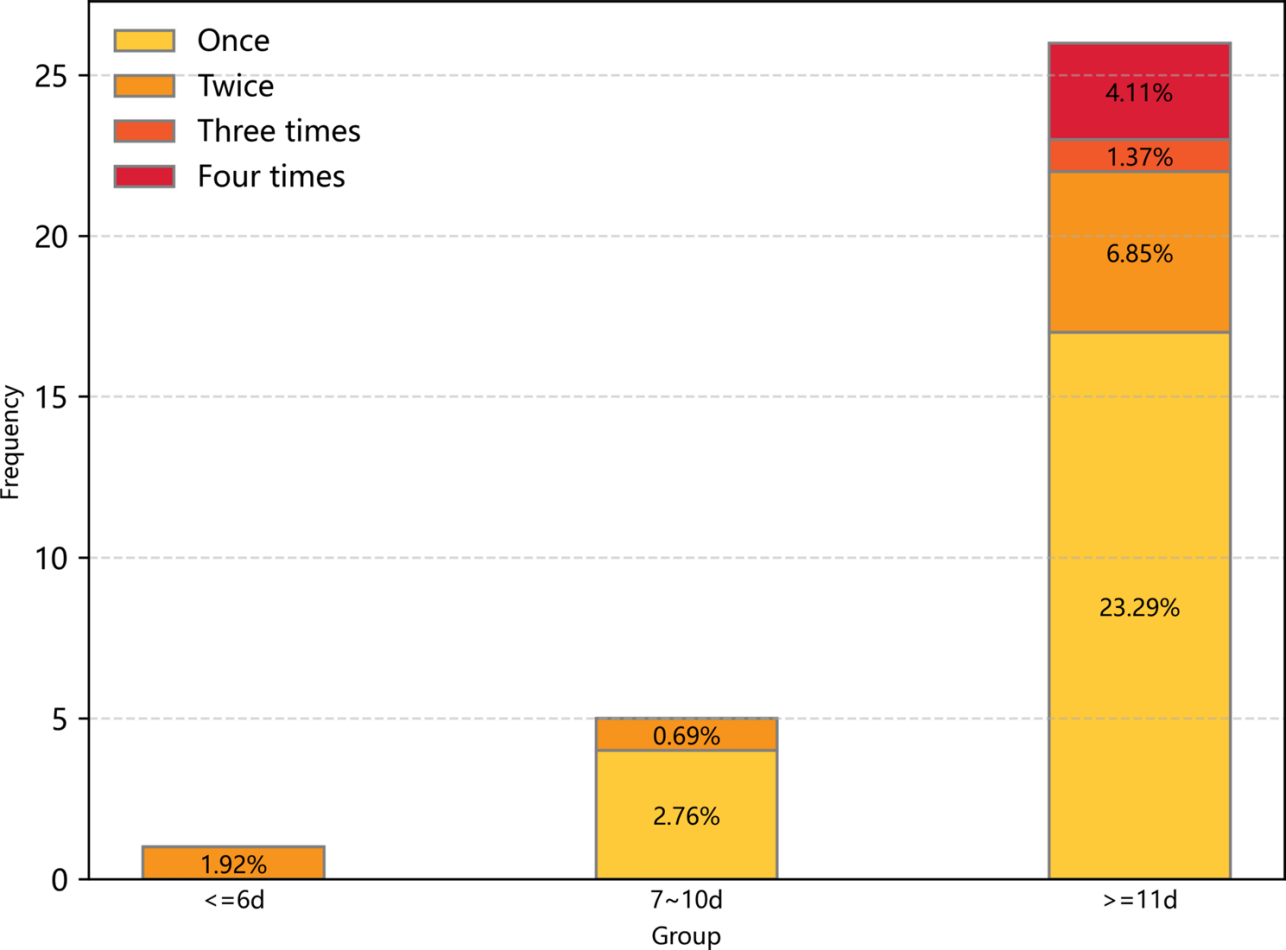
The incidence of adverse outcomes in different THD groups. Patients were classified into three groups according to the THD quartile. Frequency refers to the number of patients who experienced adverse events. Percentage refers to the incidence of adverse event. Yellow represents only one adverse event occurred, orange represents two adverse events occurred, dark orange represents three adverse events occurred, and red represents four adverse events occurred

In total, 32 (11.9%) children experienced adverse events, and the specifics are described in Additional File 3. Figure 2 visually displays the relationship between adverse outcomes and THD, showing that as THD increased, the incidence and number of adverse events also increased. We also summarized the other in-hospital outcomes in Table 4. Compared to children in the ≤ 6 days group, children in the ≥ 11 days group were associated with a higher incidence of adverse outcomes, AKI, and AHI. Additionally, the duration of mechanical ventilation, ICU stay, and hospital stay, as well as hospital costs, were significantly higher in the ≥ 11 days group.

**Table 4:** In-hospital outcomes of the study of children

Variable	Total	≤ 6 days	7 to 10 days	≥ 11 days	*P* value
Adverse event	32(11.9%)	1(1.9%)	5(3.4%)	26(35.6%)	< 0.001^b, c^
MV time (h)	12(7,21)	10(6,14)	10(6,18)	24.1(11.5,49)	< 0.001^b, c^
AKI	72(26.7%)	11(21.2%)	32(22.1%)	29(39.7%)	0.012^b, c^
AHI	62(23%)	9(17.3%)	26(17.9%)	27(37%)	0.005^b, c^
ICU stay time (d)	2(1,3)	1(1,2)	1(1,3)	4(2,6)	< 0.001^b, c^
Hospital stay time (d)	15(11,20.5)	11(9,13.8)	14(11,16)	23.4(19.29.5)	< 0.001^abc^
In-hospital cost (¥1000)	65.9(50,86.4)	60.4(48.7,72.4)	58(49.1,75.8)	95(75.5,117)	< 0.001^b, c^

Quantitative variables are expressed as median (interquartile range); Categorical variables are expressed as frequency (percentage). MV, mechanical ventilation; AKI, acute kidney injury; AHI, acute hepatic injury; ICU, intensive care unit; a: the significant difference of results between the ≤ 6 days group and the 7 to 10 days group; b: the significant difference of results between the ≤ 6 days group and the ≥ 11 days group; c: the significant difference of results between 7 to 10 days group and ≥ 11 days group

## Discussion

With the remarkable advancement of cardiac surgery technology and perioperative management in recent years, the survival rate of cardiac surgery has significantly increased [13]. This shift has redirected medical focus from reducing mortality to improving the quality of postoperative recovery and optimizing the allocation of medical resources. The mortality after cardiac surgery has become very low, leading many medical institutions to consider hospital stay duration as one of the crucial clinical indicators to evaluate postoperative recovery [14]. Early discharge and rational allocation of medical resources are currently issues of concern in the medical field. Therefore, accurately predicting discharge time and identifying high-risk patients with extended hospitalization can help arrange patients more effectively, improve bed utilization, enhance healthcare service efficiency, and optimize the rational allocation of medical resources [15]. Previous studies have employed various statistical methods to construct clinical models for predicting hospitalization duration, including regression analysis, machine learning, deep learning, etc [14–18]. However, these studies typically involve a wide range of diseases, with little attention paid to specific populations of certain heart malformations, and they often require complex statistical processes. Consequently, the use of these predictive models in clinical applications has been limited. Due to the extremely low incidence of EA, no studies have been conducted to predict the length of hospital stay for children with EA. As China’s largest cardiovascular-specialized hospital, we receive several children with EA every year. Therefore, we attempt to establish a simple model based on routinely available clinical variables to predict hospitalization duration early, utilizing preoperative and intraoperative information for EA children. In this study, univariable and multivariable COX analyses were used to identify predictors for THD in children with EA. We found that a C/*R* > 0.65, Carpentier type C or D, longer CPB time, and transfusion were associated with longer THD, whereas older age and the use of dexamethasone were associated with shorter THD.

Due to a failure of the delamination of tricuspid valve leaflets during embryonic development in EA patients, causing the posterior displacement of posterior and septal leaflets and the formation of aRV [19]. The enlargement of aRV could gradually affect the function of the functional right ventricle (fRV) and eventually result in right heart failure which would threaten the survival time for EA patients. Generally speaking, the severity of EA is inversely related to the size of fRV [20]. As the function of the right heart deteriorates, the heart enlarges, and an increased C/R is observed on chest radiographs [21]. Previous research has found that preoperative C/*R* > 0.65 is an independent predictor of poor outcomes in EA that might related to the increase in hospitalization duration [7, 22]. EA has a wide range of anatomic features due to the difference in valve leaflet displacement and the size of aRV. No classification method could cover the characteristics of all EA patients, Carpentier type [23] was widely used to categorize the anomaly of EA because of its simplicity. Despite the Carpentier type not being completely consistent with the severity of EA, Carpentier C or D usually means a larger aRV or a small fRV compared with type A or B [24]. Previous studies also have found that Carpentier C or D are usually associated with poor clinical outcomes which could prolong postoperative recovery and increase hospital stay time [20, 25–27]. In addition, CPB can cause multifaceted damage to systemic organs, including organ ischemia, reperfusion injury, activated systemic inflammatory response, oxidative stress, microemboli, and changes in cellular metabolism [28]. The direct contact of the CPB artificial pipeline with endothelial tissue activates pro-inflammatory cytokines, such as TNF-α, IL-6, TGF-β, promoting inflammatory cascade reactions and exacerbating tissue and endothelial cell damage [29]. Under the shear force caused by CPB, blood cells are prone to rupture, causing microemboli composed of fibrin, cellular fragments, fat, and air, damaging the microcirculation of organs [30]. Therefore, the longer the CPB time, the more severe the damage to the systemic system, which is not conducive to postoperative recovery. While surgeons advocate for cardiac surgery without CPB to reduce the inflammatory response and accelerate postoperative recovery, CPB is still indispensable for most pediatric complex cardiac surgeries. Moreover, a longer duration of CPB may indicate complex surgical techniques, poor patient physiology, and unexpected adverse events during surgery, all of which are related to poor prognosis and prolonged hospital stay time [31].

Blood transfusion is common during pediatric complex cardiac surgery, especially for children with low weight and preoperative anemia. However, allogeneic blood transfusion, while life-saving, may also bring negative effects, including respiratory dysfunction, kidney injury, surgical site infection, and even death [32, 33]. Besides, transfusions may be associated with other factors such as anemia, intraoperative excessive bleeding, circulatory instability, and other unexpected events that would increase the risk of prolonged THD. In our developing country, many patients are unable to undergo regular prenatal examinations due to economic constraints. EA patients often only receive surgical treatment when they present obvious symptoms such as severe tricuspid regurgitation, cyanosis, dyspnea, and right heart failure [34]. Severe EA patients are extremely critical during the infancy period and must receive surgery immediately, while mild patients only develop obvious symptoms as they grow older [19, 35]. Therefore, younger children who undergo EA corrective surgery usually present severe symptoms. Prior literature has found that compared to older children, younger children have less mature development of the circulatory and nervous systems, causing them to be susceptible to surgical and CPB stimulation, and requiring a longer time to recover [15, 36, 37]. In cardiac surgery, surgical trauma, direct contact between vascular endothelial tissue and artificial pipelines, and organ ischemia-reperfusion injury can activate systemic immune-inflammatory responses. Dexamethasone, as a glucocorticoid, can inhibit inflammatory response and reduce the associated inflammatory mediators [38]. Studies have proved that intraoperative use of dexamethasone can help reduce the duration of mechanical ventilation and hospital stay [39, 40].

Accurate prediction of hospitalization duration helps to rationally allocate medical resources, improve bed turnover, and enhance the clinical satisfaction of both patients and medical staff. Previous studies [41–43] have focused on patients in some settings: neonates, ICU, adult heart surgery, elderly stroke patients, etc. By retrospectively collecting perioperative information on these patients and utilizing relevant statistical methods, clinical models for predicting hospitalization duration are constructed. In recent years, it has become increasingly recognized that while these clinical models are effective for large populations, their predictive power for specific disease groups may be unstable. Therefore, clinical research has shifted towards focusing on patients with a single disease, aiming to construct simpler clinical models to improve their practicality [14, 15, 37]. This study only included children who underwent EA corrective surgery at our center. By collecting perioperative information, we identified relevant predictive factors for THD. In addition, we also observed that THD was associated with adverse outcomes (Fig. 2). Patients with one or more adverse outcomes often require additional medical treatment, which would prolong hospitalization duration and increase hospitalization costs. Therefore, THD can be used as an indicator to evaluate the postoperative recovery quality of patients. We identified six easily obtainable clinical variables related to THD. Clinicians can optimize perioperative clinical practices based on these variables, such as detailed preoperative discussions, improving intraoperative management strategies (including shortening CPB time, reducing allogeneic transfusion, reducing circulatory fluctuations, using anti-inflammatory drugs, etc.), and optimizing postoperative management (including infection prevention, organ protection, nutritional support, etc.). By early screening of high-risk patients of THD, clinicians could optimize perioperative management strategies, reduce adverse complications, improve postoperative recovery, and decrease medical costs.

We still recognize some limitations in our study. Firstly, this was a single-center study, which may limit the generalizability of our findings to other cardiac centers. Secondly, we only collected in-hospital outcomes and did not explore long-term outcomes. Thirdly, as we retrospectively collected the clinical data of EA patients, some clinical data may be missing. Additionally, due to the rarity of EA, the sample size was relatively small. A prospective multicenter study with a larger sample size is necessary to further explore the predictors of THD for children with EA in the future.

## Conclusions

In summary, our findings indicate that C/*R* > 0.65, Carpentier type C or D, longer CPB time, and transfusion were associated with longer THD, older age and the use of dexamethasone were associated with shorter THD. These variables are easy to obtain, clinicians can optimize perioperative medical practices and management strategies based on them. This could help in reducing adverse complications, improving postoperative recovery, and decreasing medical costs.

### Electronic supplementary material

Below is the link to the electronic supplementary material.

Supplementary Material 1

Supplementary Material 2

Supplementary Material 3

## Data Availability

The datasets generated or analyzed during the current study are not publicly available due to data protection policy in our hospital but are available from the corresponding author on reasonable request.

## Abbreviations

EA: Ebstein anomaly
THD: The Time to Hospital Discharge
aRV: Atrialized right ventricle
AHI: Acute hepatic injury
AKI: Acute kidney injury
C/R: Cardiothoracic ratio
CPB: Cardiopulmonary bypass
ICU: Intensive care unit
LVEDDz: Left ventricular end-diastolic diameter z-score
SpO2: Peripheral oxygen saturation
LCOS: Low cardiac output syndrome
fRV: Functional right ventricle
