# Supplementary material for: Predictors of length of hospital stay after pediatric Ebstein anomaly corrective surgery: a retrospective cohort study
Source: BMC Pediatr. 2024 Aug 10;24:515. doi: 10.1186/s12887-024-04936-3 (PMC11316292; doi:10.1186/s12887-024-04936-3)
Supplement: Supplementary file 1 — Supplementary Material 1 [file 12887_2024_4936_MOESM1_ESM.docx]

**Additional File** **1**

**STROBE Statement (checklist of items that should be included in reports of observational studies)**

|  | **Item**  **No.** | **Recommendation** |
| --- | --- | --- |
| **Title and abstract** | 1 | (a)Study design: a retrospective cohort study.  (b)Informative and balanced summary: The duration of a patient's hospital stay serves as a crucial indicator in evaluating postoperative recovery and surgical outcomes. This study was conducted with the objective of identifying predictors of the time to hospital discharge (THD) for children who underwent corrective surgery for Ebstein Anomaly (EA). This study included patients less than 18 years old who underwent corrective surgeries for EA. After Cox proportional hazard analysis, six predictors of THD were identified. Clinicians can utilize these variables to optimize perioperative management strategies, improving postoperative recovery, and decreasing medical costs. |
| **Introduction** |  |  |
| **Background/rationale** | 2 | Accurate prediction of hospitalization duration is crucial for hospital management and rational allocation of medical resources, which helps to improve the quality and efficiency of medical care services and avoid unnecessary long-term care. Postoperative hospital stay duration is one of the significant indicators in pediatric cardiac surgery. Even a single additional day in the hospital can significantly impact morbidity and hospital costs following cardiac surgery. However, no study explored the predictors of THD for children undergoing EA corrective surgery. |
| **Objectives** | 3 | This study aims to identify predictors of the length of hospital stay in children who underwent EA corrective surgery |
| **Methods** |  |  |
| **Study design** | 4 | A retrospective cohort study |
| **Setting** | 5 | We retrospectively collected patients(age<18y) who underwent EA corrective surgery in Fuwai hospital from January 2009 to November 2021. Data was collected from admission to discharge via an electronic medical record system. |
| **Participants** | 6 | **Inclusion criteria:**1) under 18 years old; 2) undergone EA corrective operations from January 2009 to November 2021.  **Exclusion criteria**:1) previous history of bidirectional glenn surgery; 2) complex cardiac malformations. |
| **Variables** | 7 | **Primary Outcome:** The Time to Hospital Discharge (THD), defined as the number of days from surgery to discharge.  **Secondary Outcomes:** adverse events, mechanical ventilation time, acute kidney injury, acute hepatic injury, ICU stay time, and hospitalization costs. |
| **Data sources**  **/ measurement** | 8 | **Data sources:** Electronic medical records.  **Measurement:**  THD was defined as the number of days from surgery to discharge.  LCOS was defined as a maximum vasoactive inotropic score (VISm) greater than 20 within 24 hours after surgery.  Respiratory failure was defined as a postoperative oxygenation index less than 100.  AKI was defined as a postoperative creatinine level more than 1.5 times the baseline level. AHI was defined as postoperative aspartate aminotransferase or alanine aminotransferase more than 2 times the upper limit, excluding increased AST within 24 hour. |
| **Bias** | 9 | We collected the demographics, laboratory tests, echocardiography data and surgical associated information that could affected mechanical ventilation time, we put the clinically relevant variables and variables with P<0.1 in univariable regression into multivariable regression model by forward variable selection to control the potential confounding factors. |
| **Study size** | 10 | The multivariable Cox Proportional Hazards Model typically requires a minimum of 10-15 positive events per variable, this study has a sufficient sample size for the multivariate analysis. |
| **Quantitative variables** | 11 | The THD was defined as the number of days from surgery to discharge. |
| **Statistical methods** | 12 | 1. Descriptive statistics were used to summarize the children's characteristics. Continuous data were presented as median (25th, 75th percentile) and compared using the Mann-Whitney U test (for two groups) or the Kruskal-Wallis H test (for more than two groups). Categorical variables were described as absolute counts with percentages and compared using the Chi-squared test. For the time-to-event analysis, hospital discharge was considered an event. In contrast, death before discharge was defined as a prolonged THD, input as 100 days (longer than the longest THD), and considered as no event. Univariable and multivariable COX proportional hazard models were used to assess predictors for THD. Variables with P<0.1 in the univariable model and clinically relevant variables were incorporated into a multivariable model. Collinearity relationships between predictors were evaluated by tolerance and Variance Inflation Factor (VIF). Tolerance <0.1 and/or VIF>10 indicated a collinear relationship between variables. Results from the Cox proportional hazards models are presented as hazard ratios (HRs) with 95% confidence intervals (95% CIs). HR <1 indicated a lower discharge rate (longer THD), and HR >1 indicated a higher discharge rate (shorter THD). The Kaplan‑Meier survival curves were used to present the relationships between predictors and postoperative hospital stay time. P<0.05 was considered statistically significant. All statistical analysis was conducted in SPSS software version 25 (IBM, Armonk, NY, USA) and GraphPad Prism 7.0 (GraphPad Software, Inc.). 2. There were no missing data. |
| **Results** |  |  |
| **Participants** | 13 | (a) 278 children underwent EA corrective surgeries at Fuwai Hospital over the past 13 years.  (b) 8 children were excluded due to previous history of palliative cardiac surgery(n=4), and complex cardiac malformations (n=6).  (c)Finally, 270 patients were included in this study |
| **Descriptive data** | 14 | (a) The median age of all children was 5.2(2.5,10.7) years, and 146(54.1%) were male. The median SpO2 was 98% (95%,99%), and most children combined with a heart murmur (72.6%). 19.6% (n=53) of children had a C/R>0.65 and 36 (13.3%) children with preoperative WPW syndrome. Most children had an atrial septal defect or patent foramen ovale. Regarding echocardiography results, the median ejection fraction was 65% (62%,70%), the median LVEDDz was -1.9(-2.8, -1), the median displacement of septal leaflet and posterior leaflet were 20(14,27) and 32(12,42), respectively. 180(66.7%) children presented with severe TR. Intraoperative data showed that 205(75.9%) surgeries performed modified Carpentier’s technique, the median CPB and aortic cross-clamp times were 110 (90,139) and 76 (61,96) minutes, respectively. The median VIS_m_ was 9.5(5,13), and 148(54.8%) children received transfusions.  (b)**Table 1 and Table 2** present a summary of demographic data and perioperative variables of all patients.  (c)There were no missing data. |
| **Outcome data** | 15 | (a)About the perioperative data, children in the ≥11 days group were younger, and had a lower value of BMI, hemoglobin, hematocrit, and aortic annular diameter, compared to those in the ≤6 days group. They also had higher VISm, longer CPB time, and a higher rate of C/R>0.65, Carpentier C or D, Glenn, and transfusion.  (b)About the clinical outcomes, compared to children in the ≤6 days group, children in the ≥11 days group were associated with a higher incidence of adverse outcomes, AKI, and AHI. Additionally, the duration of mechanical ventilation, ICU stay, and hospital stay, as well as hospital costs, were significantly higher in the ≥11 days group. |
| **Main results** | 16 | Upon performing a multivariable Cox analysis (Table 3), C/R>0.65, Carpentier type C or D, longer CPB time, and transfusion were still significantly associated with a lower rate of hospital discharge (i.e., longer THD), whereas older age and the use of dexamethasone were associated with a higher rate of hospital discharge (i.e., shorter THD). |
| **Other analyses** | 17 | **Figure 1** displayed the relationship of categorical variables (C/R>0.65, Carpentier type C or D, transfusion, and dexamethasone) with postoperative hospitalization.  **Figure 2** visually displays the relationship between adverse outcomes and THD, showing that as THD increased, the incidence and number of adverse events also increased. |
| **Discussion** |  |  |
| **Key results** | 18 | In this study, we retrospectively collected the perioperative data for children who underwent EA corrective surgery and used univariable and multivariable COX analyses to identify predictors for THD. We found that a Cardiothoracic Ratio (C/R) of >0.65, Carpentier type C or D, longer CPB time, and transfusion were associated with longer THD, whereas older age and the use of dexamethasone were associated with shorter THD. These variables are easy to obtain, clinicians can optimize perioperative medical practices and management strategies based on them. This could help in reducing adverse complications, improving postoperative recovery, and decreasing medical costs. |
| **Limitations** | 19 | Firstly, this was a single-center study, which may limit the generalizability of our findings to other cardiac centers. Secondly, we only collected in-hospital outcomes and did not explore long-term outcomes. Thirdly, as we retrospectively collected the clinical data of EA patients, some clinical data may be missing. Additionally, due to the rarity of EA, the sample size was relatively small. Future studies could collect more patients to further explore the relevant factors related to THD. |
| **Interpretation** | 20 | Accurate prediction of hospitalization duration helps to rationally allocate medical resources, improve bed turnover, and enhance the clinical satisfaction of both patients and medical staff. This study collecting perioperative information of children with EA and identified relevant predictive factors for THD. In addition, we also observed that THD was associated with adverse outcomes. Therefore, THD can be used as an indicator to evaluate the postoperative recovery quality of patients. We identified six easily obtainable clinical variables related to THD. Clinicians can optimize perioperative clinical practices based on these variables, such as detailed preoperative discussions, improving intraoperative management strategies (including shortening CPB time, reducing allogeneic transfusion, reducing circulatory fluctuations, using anti-inflammatory drugs, etc.), and optimizing postoperative management (including infection prevention, organ protection, nutritional support, etc.). By early screening of high-risk patients of THD, clinicians could optimize perioperative management strategies, reduce adverse complications, improve postoperative recovery, and decrease medical costs. |
| **Generalisability** | 21 | The results came from a single-center retrospective study, a prospective multicenter study with larger sample size is necessary to further explore the predictors of THD for children with EA in the future. |
| **Other information** |  |  |
| **Funding** | 22 | There were no funding supporting in this study. |
