# Supplementary material for: Predictors of length of hospital stay after pediatric Ebstein anomaly corrective surgery: a retrospective cohort study
Source: BMC Pediatr. 2024 Aug 10;24:515. doi: 10.1186/s12887-024-04936-3 (PMC11316292; doi:10.1186/s12887-024-04936-3)
Supplement: Supplementary file 2 — Supplementary Material 2 [file 12887_2024_4936_MOESM2_ESM.docx]

**Additional File 2. Univariable Cox Proportional Hazards Model**

| **Variable** | **HR(95%CI)** | **P value** |
| --- | --- | --- |
| Age (years) | 1.032(1.008,1.057) | 0.009 |
| Gestational age (weeks) | 1.027(0.922,1.145) | 0.624 |
| male | 1.126(0.884,1.433) | 0.337 |
| Weight (kg) | 1.006(1.001,1.012) | 0.022 |
| Height (cm) | 1.005(1.002,1.009) | 0.003 |
| BMI (kg/m^2^) | 1.026(0.992,1.060) | 0.131 |
| SpO2(%) | 1.024(1.005,1.043) | 0.011 |
| NYHA class (III/IV) | 0.651(0.448,0.945) | 0.024 |
| Cyanosis | 0.930(0.675,1.280) | 0.655 |
| Tachypnoea | 0.893(0.565,1.411) | 0.628 |
| Heart murmur | 0.951(0.726,1.245) | 0.713 |
| Cardiopalmus | 1.984(1.264,3.112) | 0.003 |
| Chest tightness | 1.612(0.826,3.146) | 0.162 |
| Diuretics | 1.460(0.942,2.264) | 0.100 |
| Beta-blocker | 0.675(0.332,1.372) | 0.277 |
| ACE inhibitor | 0.926(0.573,1.497) | 0.755 |
| Inotropes | 0.598(0.307,1.166) | 0.131 |
| ASD | 0.878(0.688,1.121) | 0.297 |
| PDA | 0.663(0.295,1.490) | 0.320 |
| PFO | 0.884(0.675,1.159) | 0.373 |
| WCC (10^9^/L) | 0.988(0.942,1.037) | 0.630 |
| RBC(10^12^/L) | 1.102(0.910,1.334) | 0.321 |
| PLT (10^9^/L) | 1.000(0.999,1.002) | 0.668 |
| Neutrophil | 1.027(0.964,1.094) | 0.415 |
| Lymphocyte | 0.965(0.898,1.037) | 0.329 |
| Monocyte | 0.892(0.737,1.079) | 0.240 |
| Hb(g/L) | 1.005(0.999,1.011) | 0.107 |
| Hct (%) | 1.022(1.001,1.043) | 0.038 |
| CK-MB (IU/L) | 1.001(0.998,1.004) | 0.514 |
| hs-CRP(mg/L) | 1.024(0.952,1.102) | 0.520 |
| LVEF (%) | 0.995(0.978,1.012) | 0.553 |
| TR>moderate | 1.131(0.873,1.465) | 0.350 |
| Carpentier type C or D | 0.477(0.356,0.638) | <0.001 |
| LVEDDz | 0.977(0.905,1.055) | 0.549 |
| RVAD (mm) | 0.991(0.980,1.002) | 0.095 |
| SLD (mm) | 1.003(0.994,1.012) | 0.490 |
| PLD (mm) | 1.005(0.998,1.012) | 0.195 |
| ANN (mm) | 1.030(0.995,1.065) | 0.093 |
| PVA (mm) | 1.008(0.989,1.027) | 0.425 |
| LPA (mm) | 1.034(0.988,1.082) | 0.155 |
| RPA (mm) | 1.041(0.995,1.090) | 0.082 |
| C/R>0.65 | 0.494(0.357,0.682) | <0.001 |
| WPW syndrome | 1.039(0.726,1.487) | 0.834 |
| Modified Carpentier’s method | 0.731(0.552,0.969) | 0.029 |
| Modified Danielson method | 1.374(1.033,1.826) | 0.029 |
| TVR | 0.682(0.349,1.334) | 0.264 |
| Glenn | 0.638(0.467,0.871) | 0.005 |
| Surgical ablation or division | 0.585(0.216,1.585) | 0.292 |
| Radiofrequency ablation | 0.910(0.402,2.059) | 0.821 |
| CPB time (min) | 0.995(0.992,0.998) | 0.001 |
| ACC time (min) | 0.996(0.993,1.000) | 0.028 |
| T_min_ (℃) | 1.083(0.983,1.192) | 0.107 |
| Sufentanil (ug/kg) | 1.005(0.988,1.023) | 0.554 |
| Dexamethasone | 1.494(1.156,1.930) | 0.002 |
| Ulinastatin | 0.909(0.703,1.175) | 0.466 |
| VISm | 0.983(0.966,1.000) | 0.046 |
| Infusion volume (ml) | 1.000(1.000,1.001) | 0.237 |
| Transfusion | 0.698(0.546,0.892) | 0.004 |
| Blood loss (ml) | 1.000(1.000,1.001) | 0.520 |

BMI, body mass index; SpO2, peripheral oxygen saturation; NYHA class, New York Heart Association Classification; ACEI, angiotensin-converting enzyme inhibitor; C/R, cardiothoracic ratio; WPW, Wolff-Parkinson-White; ASD, atrial septal defect; PDA, patent ductus arteriosus; PFO, patent foramen ovale; WCC, white blood cell; RBC, red blood cell; PLT, platelet; Hb, Hemoglobin; Hct, hematocrit; CK-MB, isoenzyme of creatine kinase-MB; hs-CRP, C-reactive protein; LVEF, left ventricular ejection fraction; TR, tricuspid regurgitation; LVEDDz, left ventricular end-diastolic diameter z-score; RVAD, right ventricular anteroposterior diameter; SLD, Septal leaflets displacement; PLD, Posterior leaflets displacement; ANND, aortic valve annulus; PVAD, pulmonary valve annulus diameter; LPA, left pulmonary artery diameter; RPA, right pulmonary artery diameter; TVR, tricuspid valve replacement; CPB, cardiopulmonary bypass; ACC, aortic cross-clamp; T_min_, the minimum temperature; VIS_m_, the intraoperative maximum vasoactive inotropic score;
