# Supplementary material for: Predictors of length of hospital stay after pediatric Ebstein anomaly corrective surgery: a retrospective cohort study
Source: BMC Pediatr. 2024 Aug 10;24:515. doi: 10.1186/s12887-024-04936-3 (PMC11316292; doi:10.1186/s12887-024-04936-3)
Supplement: Supplementary file 3 — Supplementary Material 3 [file 12887_2024_4936_MOESM3_ESM.docx]

**Additional File** **3. The adverse outcomes of all children**

| **Variable** | **Outcome** |
| --- | --- |
| In-hospital death | 3(1.1%) |
| Re-exploratory thoracotomy | 4(1.4%) |
| ECMO placement | 2(0.7%) |
| Reintubation | 4(1.4%) |
| Respiratory failure | 8(2.9%) |
| LCOS | 20(7.4%) |
| Malignant ventricular arrhythmia | 6(2.2%) |
| CRRT | 3(1.1) |
| Total | 32(11.8%) |

Variables are expressed as frequency (percentage); ECMO, extracorporeal membrane oxygenation; LCOS, low cardiac output syndrome; CRRT, continuous renal replacement therapy.
